# Somatostatin-expressing interneurons modulate neocortical network through GABAb receptors in a synapse-specific manner

**DOI:** 10.1038/s41598-023-35890-2

**Published:** 2023-05-31

**Authors:** Dominik Kanigowski, Karolina Bogaj, Alison L. Barth, Joanna Urban-Ciecko

**Affiliations:** 1 Laboratory of Electrophysiology, Nencki Institute of Experimental Biology, Warsaw, 02-093 Poland; 2 Department of Biological Sciences and Center for the Neural Basis of Cognition, Carnegie Mellon University, Pittsburgh, PA 15213 USA

**Keywords:** Neuroscience, Neuronal physiology, Inhibition

## Abstract

The firing activity of somatostatin-expressing inhibitory neurons (SST-INs) can suppress network activity via both GABAa and GABAb receptors (Rs). Although SST-INs do not receive GABAaR input from other SST-INs, it is possible that SST-IN-released GABA could suppress the activity of SST-INs themselves via GABAbRs, providing a negative feedback loop. Here we characterized the influence of GABAbR modulation on SST-IN activity in layer 2/3 of the somatosensory cortex in mice. We compared this to the effects of GABAbR activation on parvalbumin-expressing interneurons (PV-INs). Using in vitro whole-cell patch clamp recordings, pharmacological and optogenetic manipulations, we found that the firing activity of SST-INs suppresses excitatory drive to themselves via presynaptic GABAbRs. Postsynaptic GABAbRs did not influence SST-IN spontaneous activity or intrinsic excitability. Although GABAbRs at pre- and postsynaptic inputs to PV-INs are modestly activated during cortical network activity in vitro, the spontaneous firing of SST-INs was not the source of GABA driving this GABAbR activation. Thus, SST-IN firing regulates excitatory synaptic strength through presynaptic GABAbRs at connections between pyramidal neurons (Pyr-Pyr) and synapses between pyramidal neurons and SST-INs (Pyr-SST), but not Pyr-PV and PV-Pyr synapses. Our study indicates that two main types of neocortical inhibitory interneurons are differentially modulated by SST-IN-mediated GABA release.

## Introduction

The compound functions of the neocortex depend on neuronal microcircuits of highly interconnected glutamatergic (excitatory) neurons and GABAergic (inhibitory) interneurons. The GABAb receptors (GABAbRs) are G-coupled metabotropic receptors that are expressed pre- and postsynaptically, where they inhibit neuronal activity via the modulation of the calcium and potassium channels, respectively. GABAbRs can be found on both excitatory and inhibitory cells, however, the distribution and function of GABAbRs on specific neuronal populations and their inputs and outputs are not well-characterized, especially for molecularly and anatomically diverse inhibitory neuron subtypes that have critical roles in shaping network output. Among these interneurons, somatostatin-expressing and parvalbumin-expressing interneurons (SST-INs and PV-INs, respectively) compromise more than half of the inhibitory interneurons in the mouse neocortex^1, 2^. It has been believed that PV-INs target mainly soma, proximal parts of dendrites or the axon initial segment^3^, whereas SST-INs are considered to innervate primarily (but not exclusively) distal parts of pyramidal (Pyr) neurons^4^. Interneurons show high basal firing activity that can be regulated by brain state, behavioral tasks, and also during learning^5–7^. The activity of neocortical PV- and SST-INs is regulated in a characteristic and often opposing manner^8^. For example, in the mouse barrel cortex, L2/3 SST-INs are spontaneously active during quiet wakefulness, whereas their activity is reduced during both passive and active whisker movements^8, 9^. In contrast, PV-INs fire spontaneously during the quiet wakeful state and are profoundly activated by whisker sensing^8^. How brain state regulates the activity of these two interneurons is the subject of intense investigations.

SST-INs both directly and indirectly control neocortical network activity. Prior studies indicate that in vivo optogenetic silencing of SST-IN firing paradoxically increases the activity of neighboring Pyr neurons^8^. Our previous in vitro study identified a synaptic mechanism that may underlie this phenomenon, showing that SST-IN spontaneous activity strongly silences excitatory synaptic transmission between L2/3 Pyr neurons in mouse somatosensory cortex^10^. The effect is mediated by the presynaptic GABAbR activation, which reduces neurotransmitter release probability^11–13^. These data indicate that spontaneous activity of SST cells provides tonic inhibition to Pyr neurons, decoupling them from the network during quiet states.

SST-IN spontaneous firing may control synaptic transmission in a global- or synapse-specific manner through GABAbRs. It has been identified that GABAbRs can act as autoreceptors and suppress GABA release^14^. Most GABAergic terminals have been found to possess GABAbRs, however, little is known of GABAbR modulation in specific interneuron subtypes. Here, we compared the effects of GABAbRs on spontaneous firing, intrinsic excitability and excitatory synaptic inputs and inhibitory outputs of SST- and PV-INs in L2/3 of mouse somatosensory cortex. We also investigated how the spontaneous firing of SST-INs modulates synaptic transmission input from Pyr neurons onto these interneurons. Using in vitro electrophysiology, pharmacological and optogenetic tools, we found that SST-INs modulate neocortical network through the tonic activation of the GABAbRs in a highly synapse-specific manner.

## Results

### SST-IN spontaneous network activity is immune to tonic modulation through GABAbRs

**Figure 1:**
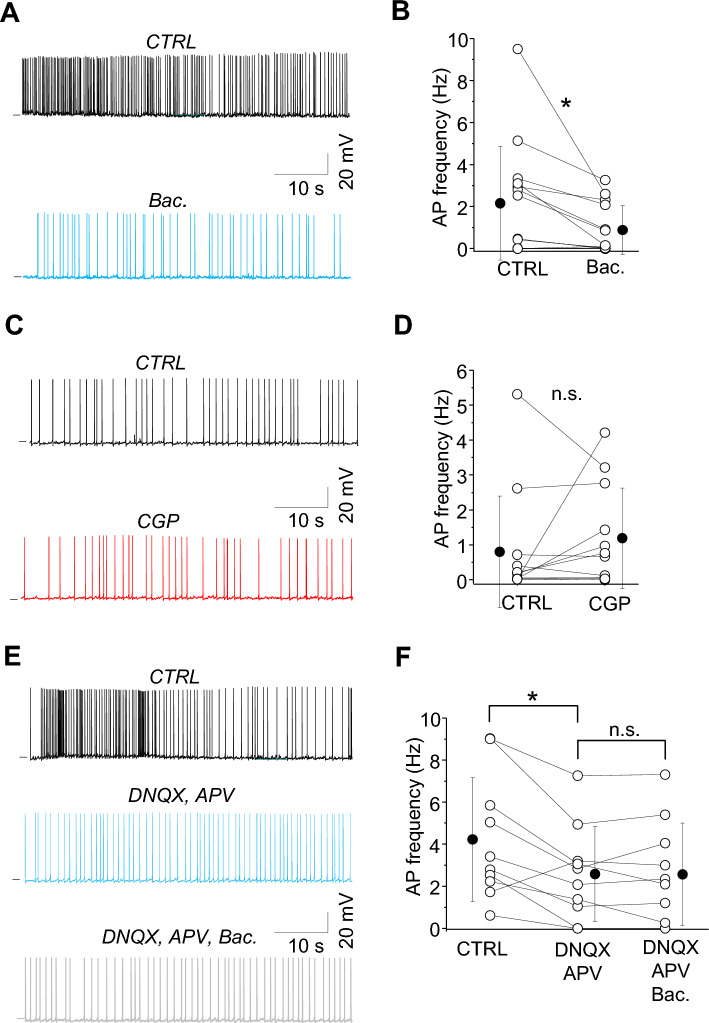
GABAbRs regulate SST-IN spontaneous activity indirectly, via suppressing excitatory drive. (**A**) Example traces of SST-IN spontaneous firing in control (CTRL) mACSF followed by the application of GABAbR agonist (baclofen, Bac.). (**B**) With-in cell comparison and mean (± SD) action potential (AP) firing of SST-INs in control and after baclofen (paired t-test,*p = 0.024, n = 14 cells). (**C**) The same as for (**A**) but for CTRL and after the application of GABAbR antagonist CGP. (**D**) With-in cell comparison and mean (± SD) action potential (AP) firing of SST-INs in two conditions showing that AP frequency does not change when GABAbR are blocked indicating that these receptors were not tonically activated in CTRL condition (paired t-test, n.s. *p =* 0.333, n = 12). (**E**) Example traces of SST-IN spontaneous firing in control mACSF (CTRL), after glutamatergic receptor antagonists (DNQX, APV) followed by the application of GABAbR agonist (Bac.). (**F**) With-in cell comparison and mean (± SD) action potential (AP) firing of SST-INs in all three conditions (paired t-test, *p = 0.006, n.s. *p =* 0.939, n = 10 cells).

SST-INs can be spontaneously active without excitatory synaptic input^15^. This spontaneous firing is linked to high levels of GABA released from SST-IN terminals and can influence GABAbRs across local networks. Here we asked whether the spontaneous activity of SST-INs might control their own activity via a feedback mechanism mediated by GABAbRs. To analyze how GABAbRs modulate activity of SST-INs we recorded their spontaneous firing in vitro using mACSF which has been known to mimic natural cerebrospinal fluid^16^ and evokes a high level of network activity^10, 17, 18^. Modified ACSF enabled us to analyze neuronal function in the condition of the background slow oscillatory activity that mimics the quiet state in vivo^19^. In this condition, L2/3 SST-INs showed elevated spontaneous firing, ranging from 0 to 9 Hz (Fig. 1) consistent with other studies^8, 20^. Bath application of the GABAbR agonist baclofen reduced the spontaneous firing of SST-INs by 60% from 2.16 ± 2.71 Hz in control to 0.88 ± 1.17 Hz in baclofen (Fig. 1A,B; n = 14, *p = 0.024, paired t-test), indicating that SST-IN activity could be influenced by GABAbRs. However, pharmacological suppression of GABAbRs did not enhance SST-IN activity, as firing rates were similar in control ACSF (0.80 ± 1.60 Hz) and after bath application of GABAbR antagonist CGP (Fig. 1C,D; 1.19 ± 1.44 Hz, n = 12, n.s. *p =* 0.333, paired t-test). Thus, under our experimental condition GABAbRs are not a prominent regulator of SST-IN activity and SST-IN spontaneous firing is not modulated by tonic activity of GABAbRs. In contrast, excitatory transmission between L2/3 pyramidal neurons is tonically suppressed by GABAbRs in this experimental condition indicating some tonic GABAbR activation in vitro^10^.

Because GABAbR agonists reduced the spontaneous firing of SST-INs, we asked whether this was via suppressing excitatory drive or decreasing the intrinsic excitability of these cells. First, we determined that excitatory synaptic drive contributes to SST-IN firing under our experimental conditions, since application of AMPA receptor and NMDA receptor antagonists (DNQX and APV) decreased spontaneous firing of SST-INs by 39% from 4.23 ± 3.0 Hz in control to 2.59 ± 2.3 Hz in antagonists (Fig. 1E,F; n = 10, *p = 0.006, paired t-test; see also^15^).

Importantly, when glutamate receptors were blocked, subsequent bath application of the GABAbR agonist baclofen did not further influence spontaneous firing frequency in SST-INs (Fig. 1E,F; 2.57 ± 2.44 Hz in baclofen, n = 10, n.s. *p =* 0.939, paired t-test). Thus, GABAbRs control SST-IN activity indirectly via the regulation of glutamatergic synaptic drive. This might be due to regulation of presynaptic release at Pyr-SST synapses, or by more complex circuit level effects^21, 22^.

**Figure 2:**
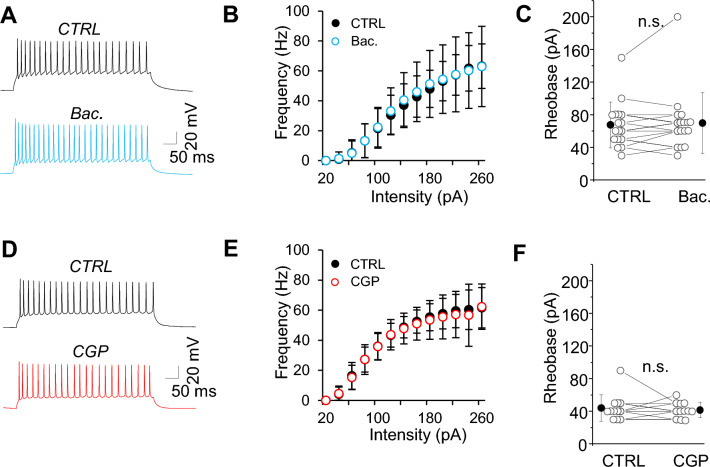
SST-IN intrinsic excitability is resistant to GABAbR modulation. (**A**) Example traces of firing responses after the somatic current injection of a 500 ms-long pulse (100 pA) in a cell recorded in CTRL rACSF followed by baclofen (Bac.). (**B**) Summary plot of the firing frequency (± SD) in response to current injections (from 0 to 270 pA) from SST-INs recorded in control ACSF and after baclofen (paired t-test, n.s., n = 17 neurons). (**C**) The comparison of mean (± SD) rheobase in control and baclofen (paired t-test, n.s. *p =* 0.553, n = 17). (**D-F**) The same as for (**A**-**C**) but for cells recorded in control mACSF followed by CGP (paired t-test, n.s. *p =* 0.571, n = 12 cells).

To confirm that GABAbRs regulate SST-IN activity only indirectly, via reducing excitatory synaptic drive to SST-INs but not the intrinsic excitability of these interneurons, we compared responses to somatic current injections before and after the application of GABAbR agonists and antagonists (Fig. 2). Neither baclofen nor CGP influenced SST-IN intrinsic excitability, because there were no differences in the I-F curve (Fig. 2B,E), rheobase current (Fig. 2C,F; in baclofen n = 17, n.s. *p =* 0.553; in CGP n = 12, n.s. *p =* 0.571, paired t-test,) and maximal frequency (in baclofen n = 17, n.s. *p =* 0.375; in CGP n = 12, n.s. *p =* 0.806, paired t-test). Also, neither input resistance nor resting membrane potentials of SST-INs were altered after drug application (Table 1). These data indicate that GABAbRs do not modulate the intrinsic excitability of SST-INs.

**Table 1:** Effects of GABAb receptor pharmacological agents on membrane properties.

	CTRL	baclofen	*p*-value (n cells)	CTRL	CGP	*p*-value (n cells)
Membrane potential (mV)						
Pyr	− 66.8 ± 4.9	− 72.5 ± 3.8	*0.0001 (10)	− 69.4 ± 4.7	− 66.4 ± 7.0	*0.04 (9)
SST	− 56.9 ± 5.3	− 58.1 ± 3.5	0.176 (11)	− 57.2 ± 4.4	− 56.2 ± 4.9	0.103 (25)
PV	− 63.1 ± 5.0	− 65.4 ± 4.5	*0.00001 (19)	− 62.9 ± 5.0	− 60.3 ± 6.3	*0.004 (15)
Input resistance (MΩ)						
Pyr	380.2 ± 60.2	229.1 ± 50.3	*0.00001 (10)	206.3 ± 37.7	247.6 ± 58.7	*0.01 (9)
SST	384.3 ± 113.5	355.1 ± 109.4	0.073 (11)	336.0 ± 106.6	332.7 ± 113.3	0.789 (22)
PV	181.0 ± 48.2	156.1 ± 43.3	*0.00005 (19)	153.9 ± 42.5	180.7 ± 52.9	*0.0008 (15)

mean ± SD.

*statistically significant.

**Figure 3:**
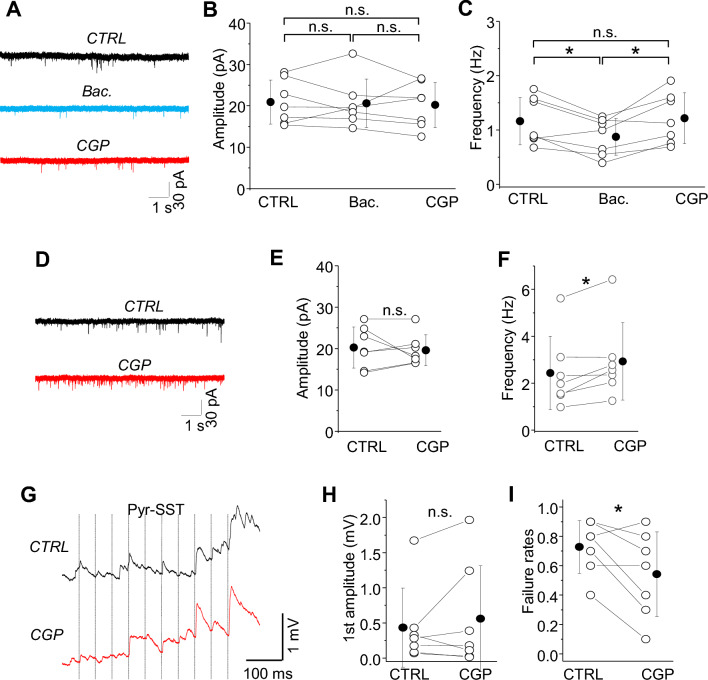
Presynaptic GABAbRs decrease the frequency of sEPSCs in SST-INs and increase the failure rates at Pyr-SST synapses. (**A**) Example traces of sEPSCs recorded in rACSF in control and after GABAbR agonist (Bac.) followed by GABAbR antagonist (CGP). (**B**) With-in cell comparison and mean (± SD) amplitude of sEPSCs in SST-INs in all three conditions (paired t-test, *p =* 0.829 ctrl vs. Bac., *p =* 0.808 Bac. vs. CGP, *p =* 0.759 ctrl vs. CGP, n = 7 cells). (**C**) The same as for (**B**) but for the frequency of sEPSCs (paired t-test, *p = 0.019 ctrl vs. Bac., *p =* 0.016 Bac. vs. CGP, n.s. *p =* 0.712 ctrl vs. CGP, n = 7 cells). (**D**) Example traces of sEPSCs recorded in mACSF in control and after GABAbR antagonist (CGP). (**E**) The mean (± SD) amplitude of sEPSCs was not different between ctrl and CGP (paired t-test, n.s. *p =* 0.664, n = 7 cells). (**F**) The mean (± SD) frequency of sEPSCs was higher in CGP than in control (paired t-test, *p = 0.019, n = 7) indicating that GABAbRs are tonically active in mACSF. (**G**) The averaged trace of 10 response trials for a synaptic connection from a L2/3 Pyr neuron to an SST-IN (Pyr-SST) under baseline condition and in the presence of the GABAbR antagonist (CGP). Ten presynaptic spikes (vertical lines) at 20 Hz were delivered. (**H**) With-in cell comparison and mean (± SD) EPSP amplitude in response to the first spike in the train, for baseline and in CGP conditions (Wilcoxon test, n.s. *p =* 0.578, n = 7 cells). (**I**) The same as for (**H**) but for failure rates (paired t-test, *p = 0.045, n = 7).

To examine whether the GABAbR-mediated suppression of SST-IN activity might occur via direct regulation of excitatory inputs onto these cells, we examined sEPSCs under low network activity conditions in regular ACSF (Fig. 3A–C). Since Pyr firing is negligible under these conditions^10, 18^, sEPSCs might be roughly equivalent to mEPSCs. The GABAbR agonist baclofen indeed suppressed the frequency of sEPSCs by 25%, from 1.16 ± 0.44 Hz in control to 0.88 ± 0.34 Hz in baclofen (Fig. 3C; n = 7, *p = 0.019, paired t-test). Consistent with the assumption that sEPSCs are equivalent to mEPSCs, baclofen had no effect on EPSC amplitude. These data suggest that GABAbRs might regulate the activity or release properties of local Pyr neurons. Application of CGP after baclofen was sufficient to restore sEPSC frequency to control values (Fig. 3A–C; 1.22 ± 0.47 Hz, n.s. *p =* 0.712, paired t-test ctrl vs. CGP). The renormalization of sEPSC frequency to control values after CGP indicates that GABAbRs are not tonically activated when the network is largely silent, consistent with our previous findings^10^.

Then, we compared these effects to conditions where network activity was enabled in mACSF, since this is associated with higher levels of GABA release from the spontaneous firing of interneurons in the cortical network^10, 18^. Bath application of CGP now increased the frequency of sEPSCs recorded in SST-INs by 20% from 2.45 ± 0.59 Hz in control to 2.94 ± 0.62 Hz in CGP (Fig. 3D–F; n = 7, *p = 0.019, paired t-test) but not the sEPSC amplitude (Fig. 3E; 20.29 ± 1.87 pA in control and 19.63 ± 1.42 pA in CGP, n.s. *p =* 0.664, paired t-test).

In summary, these experiments suggest that GABAbRs do not directly influence SST-IN activity through cell-autonomous mechanisms, but rather through regulation of excitatory inputs. The change in sEPSC frequency but not amplitude are consistent with modulation of presynaptic release properties at Pyr-SST synapses^10, 18^, although we cannot exclude other indirect network effects. The absence of GABAbR modulation of SST-IN membrane properties and intrinsic excitability suggests that SST-IN activity may regulate network function independently, without a negative feedback loop. To these ends, it is significant that SST neurons do not synapse onto each other, like other interneurons subtypes in the cortical network^23^.

### SST-INs suppress L2/3 Pyr-SST synapses through GABAb receptors

L2/3 Pyr neurons are densely connected to nearby SST-INs in primary sensory cortex (rev.;^23^), although synaptic transmission from these connections is generally weak^18, 24–26^. Excitatory connections between Pyr neurons can be silenced by presynaptic GABAbRs, and the spontaneous firing of SST-INs is sufficient to suppress Pyr to Pyr (Pyr-Pyr) communication^10^. Here, we decided to examine whether spontaneous activity of SST-INs could also regulate excitatory drive onto SST-INs themselves. This is important, because it would indicate that when SST-IN activity is high (perhaps due to control by neuromodulators) they are unresponsive to sensory drive or local computations.

First we determined whether L2/3 Pyr-SST synapses could be regulated by GABAbRs. Using dual patch-clamp recording, we established synaptically connected Pyr-SST pairs in L2/3 of the somatosensory cortex in a mouse brain slice in mACSF (Fig. 3G–I). Bath application of the GABAbR blocker CGP reduced failure rates of Pyr-SST EPSPs by 26% (Fig. 3I; from 0.73 ± 0.18 to 0.54 ± 0.29; n = 7, *p = 0.045, paired t-test) but did not change EPSP amplitude, consistent with the low release probability at these synapses (Fig. 3H; 0.43 ± 0.56 mV in control and 0.56 ± 0.75 mV in CGP, n.s. *p =* 0.578, Wilcoxon test).

**Figure 4:**
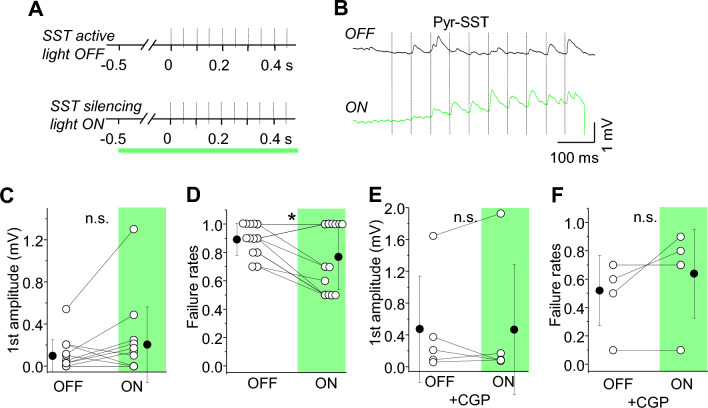
Somatostatin cell silencing enhances EPSP efficacy by reducing failure rates. (**A**) Schematic of the stimulation protocol. 1-single green light (1 s) was started 0.5 s prior to the presynaptic spike train. (**B**) The averaged trace of EPSP under baseline/light OFF and light ON conditions. (**C**) With-in cell comparison and mean (± SD) EPSP amplitude in response to the first spike in the train, for both conditions (paired t-test, n.s. *p =* 0.15, n = 13 cells). (**D**) With-in cell comparison and mean (± SD) failure rates after the first spike, for both conditions (paired t-test, *p = 0.022, n = 13). (**E**) and (**F**) The same as (**C**) and (**D**) but in the presence of the GABAb receptors antagonist (paired t-test, n.s. *p =* 0.994, *p =* 0.208, n = 5 cells).

To check whether SST-IN firing can also regulate Pyr-SST synapses, we optogenetically suppressed SST-IN spontaneous activity (Fig. 4) using transgenic expression of the hyperpolarizing pump (Arch) in SST-Cre mice. Using paired whole-cell recordings of synaptically connected Pyr-SST, we tested whether acute silencing of SST-IN spontaneous activity might enhance EPSP strength and reliability of these connections. Illumination of the brain slice with yellow-green light (LED) for 1–1.5 s fully suppressed SST-IN firing, hyperpolarizing their membrane potentials by about 10–20 mV. During the light ON period, Pyr-SST EPSP failure rates were significantly reduced compared to OFF trials (Fig. 4D; 0.89 ± 0.11 mV in OFF and 0.77 ± 0.23 mV in ON, n = 13, *p = 0.022, paired t-test) and EPSP amplitude was not changed (Fig. 4C; 0.10 ± 0.15 mV in OFF and 0.20 ± 0.36 mV in ON, n = 13, n.s. *p =* 0.15, paired t-test). Thus, the effect of SST-IN silencing is consistent with the pharmacological results of GABAbR manipulation, where EPSP failure rates were reduced in the presence of GABAbR antagonists (Fig. 3G–I).

To confirm that this effect was due to GABAbRs, we analyzed the effect of SST-IN silencing when GABAbRs were blocked by CGP (Fig. 4E,F). Under these conditions, SST cell silencing did not change EPSP failure rates (Fig. 4F; 0.52 ± 0.25 mV in OFF and 0.64 ± 0.31 mV in ON, n.s. *p =* 0.208, paired t-test) or amplitude (Fig. 4E; 0.48 ± 0.67 mV in OFF and 0.47 ± 0.82 mV in ON, n = 5, n.s. *p =* 0.994, paired t-test), indicating that the effect was fully mediated by these receptors.

### No tonic activation of presynaptic GABAbRs at SST-IN output onto Pyr neurons

GABA released from SST-INs drives fast, GABAaR-mediated, synaptic inhibition of postsynaptic neurons. The activation of presynaptic GABAbRs at inhibitory terminals (so-called autoreceptors) can suppress GABA release and thus reduce synaptic inhibition^12^. Do SST-INs also possess presynaptic GABAbRs at synapses onto Pyr neurons? SST-INs to Pyr (SST-Pyr) connections are very common, where the L2/3 connection probability reached 60% (45 connected pairs out of 75 tested), consistent with other reports^27, 28^.

**Figure 5:**
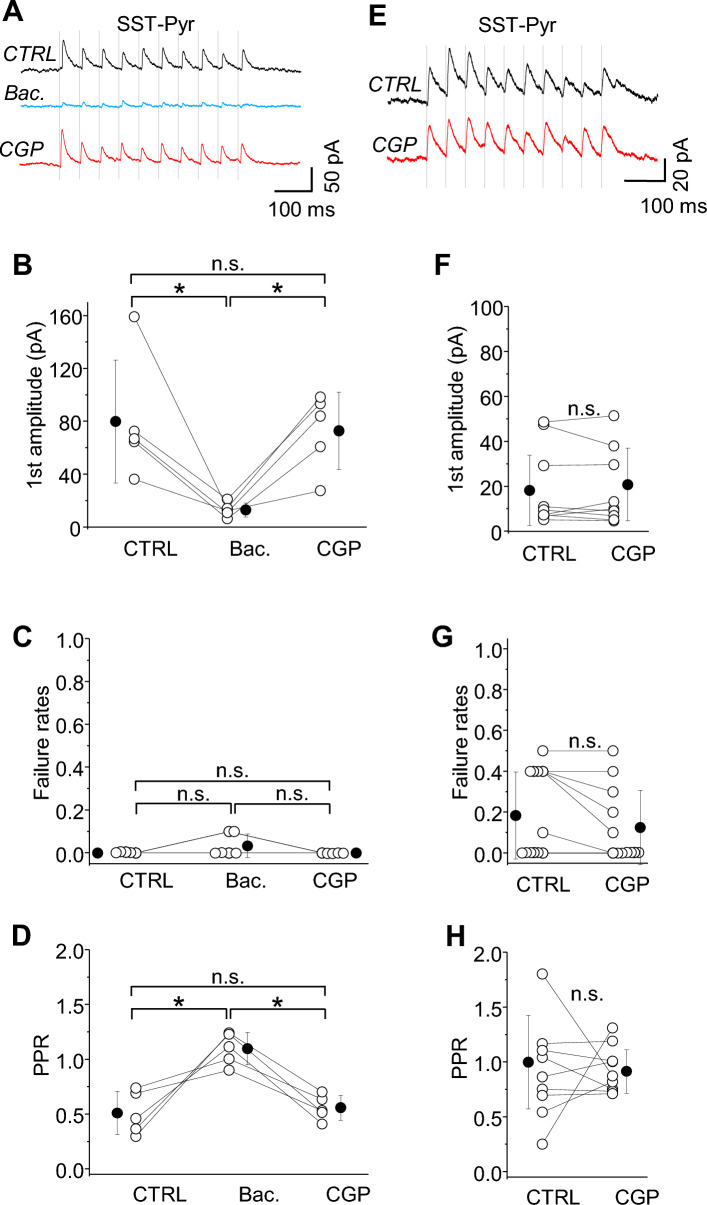
SST-IN outputs to Pyr neurons are not regulated by tonic activity of GABAbRs. (**A**) The averaged trace of 10 response trials for a paired recording of a synaptic connection from an SST-IN to a L2/3 Pyr neuron (SST-Pyr) in control rACSF followed by baclofen and CGP. Ten presynaptic spikes (vertical lines) at 20 Hz were delivered. (**B**) With-in cell comparison and mean (± SD) IPSC amplitude in response to the first spike in the train, for control, baclofen and in CGP conditions (paired t-test, *p = 0.031 ctrl vs. Bac.; *p = 0.008 Bac. vs. CGP; n.s. *p =* 0.654 ctrl vs. CGP; n = 5). (**C**) The same as for (**B**) but for failure rates (paired t-test, n.s. *p =* 0.178 ctrl vs. Bac; n.s. *p =* 0.178 Bac. vs. CGP; Wilcoxon test, n.s. *p =* 0.5 ctrl vs. CGP; n = 5). (**D**) The same as for (**B**) but for PPR (paired t-test, *p = 0.017 ctrl vs. Bac; *p = 0.002 Bac. vs. CGP; Wilcoxon test, ctrl vs. CGP n.s. *p =* 0.5; n = 5). (**E**) The averaged trace of 10 response trials for a paired recording of a synaptic connection from an SST-IN to a L2/3 Pyr neuron (SST-Pyr) in control mACSF and in CGP. Ten presynaptic spikes (vertical lines) at 20 Hz were delivered. (**F**) With-in cell comparison and mean (± SD) IPSC amplitude in response to the first spike in the train, for control and in CGP conditions (paired t-test, n.s. *p =* 0.252, n = 12 cells). (**G**) The same as for (**F**) but for failure rates (Wilcoxon test, n.s. *p =* 0.125, n = 12 cells). (**H**) The same as for (**F**) but for PPR (paired t-test, n.s. *p =* 0.558, n = 12 cells).

Using paired whole-cell recordings of connected SST and Pyr neurons (SST-Pyr), we compared the effects of GABAbR agonist (baclofen) on IPSC. Bath application of baclofen decreased the first IPSC amplitude by 84% (Fig. 5A,B; control 79.94 ± 46.49 pA vs. baclofen 13.09 ± 5.35 pA; n = 5, *p = 0.031, paired t-test) but had no effect on failure rates (Fig. 5C; 0.00 ± 0.00 in control to 0.04 ± 0.05 in baclofen, n.s. *p =* 0.5, Wilcoxon test). Wash-on of CGP reversed the effects on IPSC amplitude to control values (Fig. 5B; 72.87 ± 29.12 pA in CGP, n.s. *p =* 0.654 ctr vs. CGP, paired t-test). There was no effect on failure rates, since synaptic efficacy was high at these connections (Fig. 5C; CGP 0.0 ± 0.0, n.s. *p =* 0.5, ctrl vs. CGP, Wilcoxon test). If there are presynaptic GABAbRs at SST-IN terminals, we predicted that the GABAbR agonist baclofen would increase the paired-pulse ratio of the second response to the first (PPR) at SST-Pyr synapses. In superficial layers of the neocortex, SST-Pyr synapses are typically depressing, but this was shifted to a mild facilitation after the application of baclofen (Fig. 5D; control 0.51 ± 0.20 versus baclofen 1.10 ± 0.15; *p = 0.017, paired t-test), indicating a presynaptic locus of drug action. Subsequent bath application of the GABAbR antagonist CGP reversed this facilitation back to control levels (Fig. 5D; 0.56 ± 0.11 in CGP, n.s. *p =* 0.661, paired t-test), suggesting that GABAbR activation was negligible under our recording conditions and consistent with the results obtained for SST-INs in the hippocampus^29^.

Because the agonist was effective at reducing neurotransmitter release at SST-Pyr synapses, we asked whether presynaptic GABAbRs might sometimes be activated by the synapse’s own GABA release. In this case, the amplitude of the IPSC might be suppressed when neocortical SST neurons fire at some regular frequency, as has been described in vivo during the quiet resting state^8, 20^ and also in vitro, in mACSF^10, 15, 18^. We thus examined the IPSC of connected SST and Pyr neurons in mACSF (Fig. 5E–H). Under baseline conditions, the evoked IPSC had a relatively low failure rate (0.18 ± 0.21, n = 12, Fig. 5G) indicating that GABAaR-dependent inhibition from SST-INs is strong even when spontaneous network activity is high. Bath application of CGP did not alter IPSC amplitude (Fig. 5F; control 18.28 ± 15.65 pA vs. CGP 20.82 ± 16.08 pA, n = 12, n.s. *p =* 0.252, paired t-test), failure rates (Fig. 5G; control 0.18 ± 0.21 vs. CGP 0.13 ± 0.18, n.s. *p =* 0.125, Wilcoxon test) or PPR (Fig. 5H; control 1.00 ± 0.43 vs. CGP 0.92 ± 0.20, n.s. *p =* 0.558, paired t-test). Altogether, these data indicate that although SST-INs have presynaptic GABAbRs, spontaneous network activity may not be sufficient to activate these receptors.

Importantly (and in contrast to Pyr neurons), we did not observe any changes in resting membrane potential nor input resistance of SST-INs when GABAbRs were either activated or blocked pharmacologically (Table 1). These data indicate that GABAbRs in neocortical SST-INs are unlikely to act through potassium channels, similarly what has been observed for SST-INs in the hippocampal network^30^.

To confirm that the spontaneous activity of SST-IN is not sufficient for the tonic activation of GABAbRs at SST-Pyr synapses, we optogenetically silenced SST-INs to check whether it influences SST-Pyr connections (Supplementary Fig. 1). Analysis of SST-Pyr synapses showed that SST-IN silencing changed neither IPSC amplitude (Supplementary Fig. 1A–C; OFF 28.31 ± 21.74 pA vs. ON 28.91 ± 25.59, n = 5, n.s. *p =* 0.787, paired t-test), nor failure rates (Supplementary Fig. 1D; 0.08 ± 0.13 in OFF and 0.10 ± 0.12 in ON, n.s. *p =* 0.374, paired t-test) nor PPR (Supplementary Fig. 1E; 0.79 ± 0.38 in OFF and 0.74 ± 0.11 in ON, n.s. *p =* 0.827, paired t-test). Thus, direct GABAaR-mediated inhibition from SST-INs is not influenced by presynaptic GABAbRs during spontaneous activity of SST-INs.

### GABAbRs modulate intrinsic excitability of L2/3 PV-INs in the neocortex

**Figure 6:**
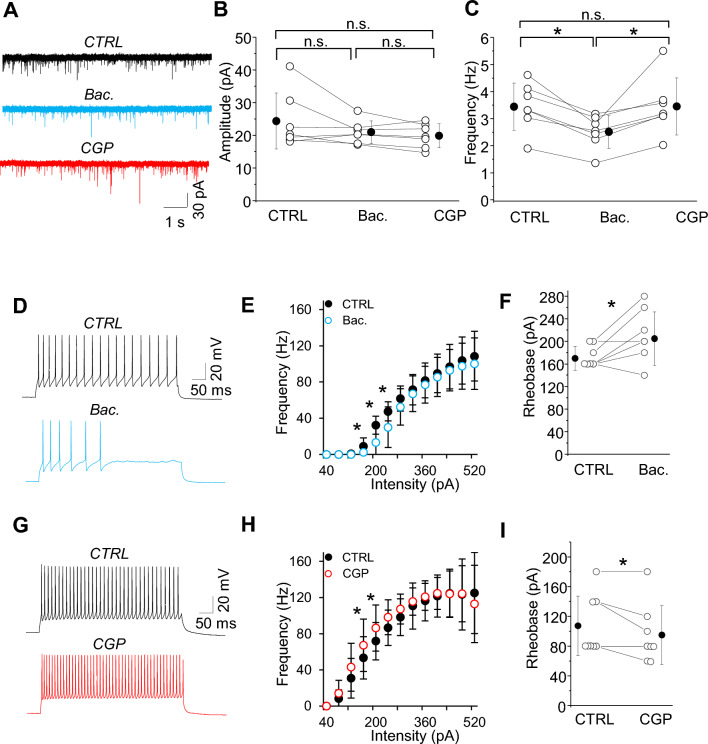
GABAbR modulation of excitatory synaptic drive to PV-INs and their intrinsic excitability. (**A**) Example traces of sEPSCs recorded in rACSF in control and after GABAbR agonist (Bac.) followed by GABAbR antagonist (CGP). (**B**) With-in cell comparison and mean (± SD) amplitude of sEPSCs in PV-INs in all three conditions (paired t-test, n.s. *p =* 0.168 ctrl vs. Bac., n.s. *p =* 0.207 Bac. vs. CGP, n.s. *p =* 0.139 ctrl vs. CGP, n = 7 cells). (**C**) The same as for (**B**) but for the frequency of sEPSCs (paired t-test, *0.001 ctrl vs. Bac., 0.019 Bac. vs. CGP, n.s. 0.944 ctrl vs. CGP, n = 7 cells) (**D**-**I**) The effect of GABAbR pharmacological manipulations on PV-IN intrinsic excitability. (**D**) and (**G**) Example traces of firing responses after the somatic current injection of a 500 ms-long pulse (200 pA) in two separate cells recorded in control followed by baclofen (Bac.) and control followed by CGP, respectively. (**E**) Summary of the firing frequency (± SD) in response to current injections (from 0 to 550 pA) from PV-INs recorded in control ACSF and after baclofen (paired t-test, n = 11). (**H**) The same as for (**E**) but for CGP (paired t-test, n = 8 cells).

Does the spontaneous activity of SST-IN activate GABAbRs on other neocortical neurons? Because PV neurons are a prominent source of inhibition in cortical networks^26, 27, 31, 32^, we examined the effect of GABAbR pharmacological modulation on both the intrinsic excitability of PV-INs (Fig. 6D–I) as well as Pyr to PV-IN inputs in L2/3 (Fig. 6A–C). GABAbR activation using baclofen reduced the frequency of sEPSCs measured in PV-INs by 27% (Fig. 6A–C; control 3.45 ± 0.87 Hz vs. baclofen 2.52 ± 0.61 Hz, n = 7, *p = 0.001, paired t-test) but did not alter sEPSC amplitude (Fig. 6A–C; control 24.38 ± 8.52 pA vs. baclofen 20.96 ± 3.48 pA, n.s. *p =* 0.168, paired t-test). CGP fully reversed these effects (Fig. 6A–C; amplitude in CGP 19.86 ± 3.66 pA, n.s. *p =* 0.138; frequency in CGP 3.46 ± 1.05 Hz, n.s. *p =* 0.944, ctrl vs. CGP, paired t-test). Since changes in the event frequency are associated with changes in neurotransmitter release, this result suggests that presynaptic GABAbR at Pyr-PV synapses can modulate excitatory synaptic transmission to L2/3 PV-INs.

Next, we analyzed PV-IN intrinsic excitability. In contrast to SST-INs, pharmacological activation of GABAbRs profoundly influenced the intrinsic membrane properties of PV neurons (Fig. 6D–I). Bath application of baclofen suppressed the I-F curve (Fig. 6D,E), increased rheobase current (Fig. 6F, control 170 ± 21.38 pA vs. baclofen 205 ± 47.51 pA, n = 8, *p = 0.031, paired t-test) and decreased maximal firing frequency (control 128 ± 36.41 Hz vs. baclofen 113 ± 32.07 Hz, n = 8, *p = 0.008, Wilcoxon test, data not shown). CGP had the opposite effect, increasing evoked spiking with current injection (Fig. 6G,H), decreasing rheobase current (Fig. 6I; control 107.50 ± 39.90 pA vs. CGP 95.00 ± 39.64 pA, n = 8, *p = 0.049, paired t-test,), although there was no effect on maximal firing frequency (control 132.00 ± 40.95 Hz vs. CGP 135.00 ± 23.77 Hz, n.s. *p =* 0.816, paired t-test, data not shown). Thus, unlike SST-INs, spontaneous network activity is associated with tonic GABAbR activation in PV-INs that significantly suppresses the activity of these interneurons.

The reduction in PV-IN excitability was associated with significant GABAbR modulation of Vrest. Resting membrane potential was hyperpolarized by ~ 2 mV by baclofen and depolarized by ~ 2 mV with CGP, and input resistance was decreased by baclofen and increased with CGP (Table 1). In contrast, both parameters in SST-INs showed no significant difference between control and drug applications (Table 1). These data suggest that postsynaptic GABAbRs are coupled to potassium channels in PV- but not SST-INs^30^.

### GABAbR-mediated suppression of PV-IN output onto Pyr neurons

**Figure 7:**
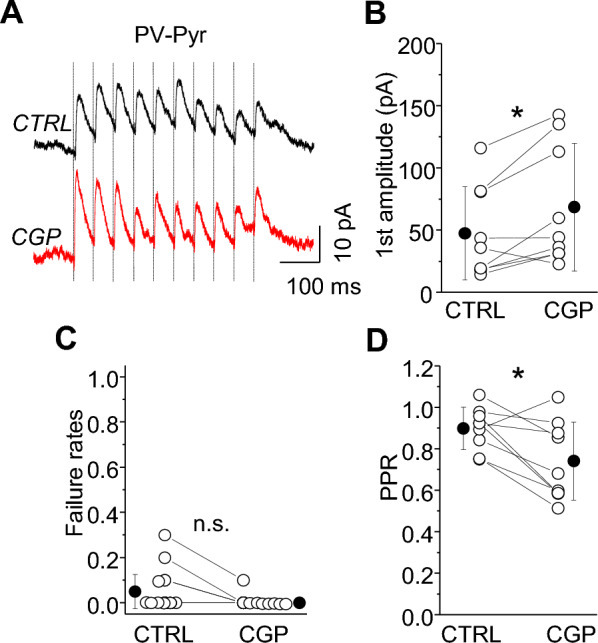
PV-Pyr synapses are regulated by tonic activity of GABAbRs. (**A**) The averaged trace of 10 response trials for a synaptic connection from a PV-IN to a L2/3 Pyr neuron (PV-Pyr) under baseline condition and in the presence of the GABAbR antagonist (CGP). Ten presynaptic spikes (vertical lines) at 20 Hz were delivered. (**B**) With-in cell comparison and mean (± SD) IPSC amplitude in response to the first spike in the train, for baseline and in CGP conditions (paired t-test, *p = 0.035, n = 8). (**C**) With-in cell comparison and mean(± SD) failure rates after the first spike, for both conditions (Wilcoxon test, n.s. *p =* 0.250, n = 8). (**D**) The same as for (**C**) but for PPR (paired t-test, n.s. *p =* 0.043, n = 8).

GABAbRs on PV-INs might primarily control the intrinsic membrane properties of these cells, or they also can directly regulate synaptic release. To determine whether L2/3 PV-IN terminals have presynaptic GABAbRs, we examined the effects of GABAbR antagonist on PV-IN-mediated IPSCs in Pyr neurons using paired whole-cell recordings (Fig. 7). PV-Pyr connections are extremely abundant in L2/3^1, 28^, and we also observed that the probability of PV-Pyr connections reached 82% (9 connected pairs out of 11 tested). When network activity was high (in mACSF), we observed that IPSC failure rates were very low (0.05 ± 0.08 pA, n = 8, Fig. 7C), indicating that the efficacy of PV-Pyr synapses was very high even in the presence of high spontaneous firing of SST-INs. Bath application of the GABAbR antagonist CGP increased IPSC amplitude by 48% (Fig. 7A,B; control 51.09 ± 37.44 pA vs. CGP 69.70 ± 51.36 pA, n = 8, *p = 0.035, paired t-test). Because failure rates were already negligible, CGP had no effect (Fig. 7C; 0.00 ± 0.00 in CGP, n.s. *p =* 0.250, Wilcoxon test). PPR decreased from 0.89 ± 0.11 in control to 0.76 ± 0.19 in CGP (Fig. 7D; n = 8, *p = 0.043, paired t-test). The effects of CGP on IPSC amplitude and PPR indicate that fast inhibition from PV-INs is modulated by tonic activity of presynaptic GABAbRs under spontaneous network activity in acute brain slices.

### Tonic GABAbR activity in PV-INs is not regulated by spontaneous activity of SST-INs

SST-INs inhibit cortical networks directly, via fast synaptic transmission. In addition, through their spontaneous activity they also suppress communication between Pyr neurons via GABAbRs^10^. To determine whether the spontaneous activity of SST-INs can activate GABAbRs at synapses onto and from PV-IN, we crossed PV-Tdt mice with SST-Cre mice and virally transduced ArchT into SST cells.

**Figure 8:**
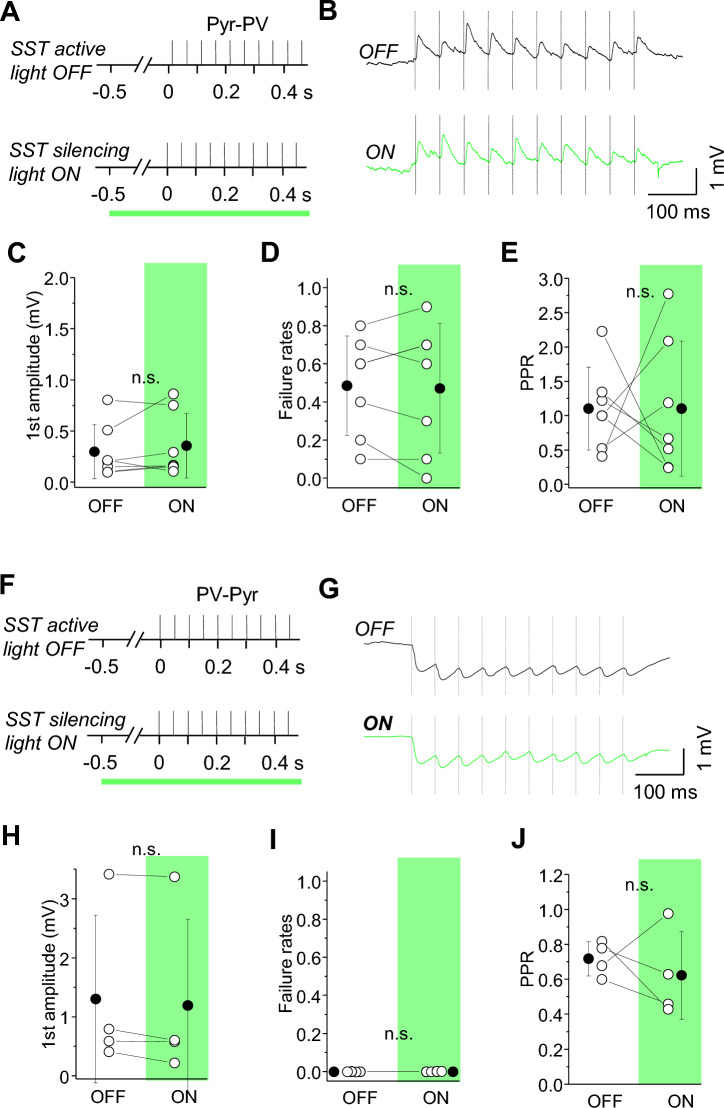
SST-IN spontaneous firing does not control inputs and outputs of PV-INs. (**A**) ArchrhodopsinT was expressed in SST-INs and Pyr to PV EPSP were analyzed in paired recordings. Schematic of the stimulation protocol. 1-single green light (1 s) was started 0.5 s prior to the presynaptic spike train. (**B**) An example of the averaged trace of EPSP under baseline/light OFF and light ON conditions. (**C**) With-in cell comparison and mean (± SD) EPSP amplitude in response to the first spike in the train, for baseline and light ON conditions (paired t-test, n.s. *p =* 0.337, n = 7). (**D**) With-in cell comparison and mean (± SD) EPSP failure rates for baseline and light ON conditions (paired t-test, n.s. *p =* 0.766, n = 7). (**E**) The same as for (**D**) but for PPR (paired t-test, n.s. *p =* 0.999, n = 7). (**F**-**J**) The same as for (**A**-**E**) but for IPSPs recorded in PV to Pyr connections (n.s. *p =* 0.107, *p =* 1, *p =* 0.554, respectively, n = 4).

Using dual patch-clamp recordings, we tested whether acute silencing of SST-INs could change EPSP strength and reliability at both Pyr-PV and PV-Pyr synapses. SST-INs were silenced for 1 s before activation of Pyr-PV synapses (Fig. 8A,B). Surprisingly, SST-IN silencing had no effect on EPSP amplitude at Pyr-PV connections (Fig. 8C; OFF 0.30 ± 0.26 vs. ON 0.36 ± 0.32 mV, n = 7, n.s. *p =* 0.337, paired t-test). Also, failure rates and PPR at Pyr-PV synapses were not statistically different in light OFF and ON conditions (Fig. 8D,E; failure rates 0.49 ± 0.26 in OFF and 0.47 ± 0.34 in ON; PPR 1.10 ± 0.06 in OFF and 1.10 ± 0.98 in ON, n.s. *p =* 0.766 and *p =* 0.999, respectively). Similarly, IPSP amplitude, failure rates and PPR at PV-Pyr connections were not changed when SST-IN activity was optogenetically suppressed (Fig. 8F–J). IPSP amplitude was 1.30 ± 0.42 mV in light OFF and 1.20 ± 1.46 mV in light ON (Fig. 8H; n = 4, n.s. *p =* 0.107, paired t-test), failure rates were 0.00 ± 0.00 in OFF and ON conditions (Fig. 8I; n.s. *p =* 1, paired t-test) and PPR was 0.72 ± 0.10 in OFF and 0.62 ± 0.25 in ON (Fig. 8J; n.s. *p =* 0.554, paired t-test). These findings demonstrate that despite the presence of GABAbRs at excitatory synapses onto PV-INs as well as PV-INs outputs onto Pyr cell targets, SST-IN spontaneous firing is not sufficient to regulate these connections.

## Discussion

GABAbRs regulate both synaptic transmission as well as membrane potential, and the tonic activation of GABAbRs can regulate synaptic function and overall network activity. Prior studies have shown that SST neurons are an important source of GABA that activates GABAbRs^10^. Here we investigate whether SST neurons themselves might be regulated by GABAbRs as a possible negative feedback loop in the cortical circuit using targeted whole-cell patch clamp recordings in acute brain slices. Pharmacological activation of GABAbRs reduced excitatory drive onto SST-INs to decrease SST-IN spontaneous activity, and optogenetic suppression of SST-IN activity was sufficient to enhance synaptic transmission at Pyr-SST synapses in a GABAbR-dependent manner. These data suggest that SST-IN firing is part of a negative feedback loop, by which the activity of SST-INs suppresses the effect of local excitatory input in further activating SST-INs. However, under network-active conditions where SST neurons are depolarized and their firing activity is high^8, 10, 15, 19^, GABAbR antagonist application did not further enhance SST-IN firing activity. These results suggest that ambient GABA from spontaneous network activity may not be sufficient to regulate SST-IN firing output. In contrast, ambient GABA during spontaneous network activity could regulate synaptic properties of both excitatory inputs onto PV-INs as well as PV-IN synaptic output via GABAbRs. GABAbRs also controlled the intrinsic excitability of PV-INs. However, we determined that this tonic GABAbR activation was unlikely to come from SST-INs, since optogenetic silencing of SST neurons did not influence PV-IN-related synapses.

Altogether, our data indicate that GABAbRs modulate the activity of SST- and PV-INs in a different way. Under conditions of elevated spontaneous network activity, excitatory synaptic inputs to both SST- and PV-INs are depressed by tonic activity of GABAbRs, reducing the influence of local excitatory drive onto these inhibitory neurons to diminish network inhibition. This effect is enhanced by the fact that PV-IN-mediated feedback inhibition onto Pyr neurons is markedly reduced by the tonic activation of GABAbRs.

Both light and electron microscopic analysis of GABAbR immunoreactivity shows that these receptors are expressed in cell bodies, dendrites and terminals of most types of neurons in the neocortex and the hippocampus; however, the net effect of GABAbRs will depend upon the specific ion channels that they are linked to, leading to different effects on network function and connectivity. Canonically, postsynaptic GABAbRs activate G-protein-coupled inwardly-rectifying potassium channels (such as Kir-3), leading to hyperpolarization of the neuronal membrane and thus decreasing neuronal activity^33–35^. However, consistent with the analysis of hippocampal SST-INs^30^, we observed a moderate but not significant alteration in membrane potential and input resistance of L2/3 SST-INs after GABAbR agonist and antagonist administration, suggesting that neocortical SST-INs possess postsynaptic GABAbRs which are not strongly coupled to Kir channels. It is possible that GABAbRs might be co-clustered with and inhibit L-type channels as observed in hippocampal SST-INs^30^. In contrast to SST-INs, we found significant changes in the membrane potential and input resistance in L2/3 PV-INs and Pyr after the application of GABAbR agonists and antagonists, indicating that postsynaptic GABAbRs activate potassium channels on these neurons. Thus, the cell-type specific differences in pharmacological effects on resting membrane potential and input resistance between these cell types are due to the localization of Kir channels that are sensitive to GABAbR modulation.

Presynaptic GABAbRs, which can suppress the release of both glutamate and GABA, are widely distributed across the brain. However, it is unclear whether specific interneuron subtypes possess GABAbRs that modulate synaptic release. Immunoreactivity for presynaptic GABAbRs has been observed on hippocampal interneurons expressing SST, neuropeptide Y, calretinin, calbindin, or cholecystokinin (CCK) but not on PV-INs^36^. Other studies that used electrophysiological recordings and freeze-fracture replica-based quantitative immunogold electron microscopy have revealed the presence of presynaptic GABAbRs on hippocampal CCK and PV-INs^14^. Fast, GABAaR inhibition from PV to Pyr neurons is weakly reduced by presynaptic GABAbRs in the hippocampus^14, 37, 38^; however, these effects may be region-specific since GABAa-mediated inhibition from L5 fast-spiking (presumably PV) interneurons was strongly modulated by GABAbRs^39^. In developing gerbil auditory cortex, GABAaR-mediated inhibition from L2/3 FS (presumably PV-INs), but not from LTS (presumably SST-INs) is controlled by presynaptic GABAbRs^40^. Our study showed that despite the fact that both SST- and PV-INs have presynaptic GABAbRs (autoreceptors), only the fast inhibition from PV-INs is tonically suppressed through GABAbRs, whereas fast inhibition mediated by SST-INs appears insensitive to GABAbR tonic modulation. Thus, mechanisms for presynaptic GABAbR modulation are region-specific, differing between hippocampal and neocortical interneurons.

In vitro recordings show that SST-INs can exhibit high levels of spontaneous activity that is independent of glutamatergic input or electrical stimulation^15^. Indeed, we find that local synaptic drive to SST-INs under our in vitro recording conditions is markedly low, consistent with ours and others’ previous work rev.;^23, 41^. Here we show that optogenetic silencing of SST-INs in these active network states enhanced synaptic input from Pyr to SST-INs, an effect that was dependent upon GABAbRs. These data suggest that SST-IN inhibition may exist in two different regimes: one, where spontaneous SST-IN activity is temporally imprecise and has a more global effect on reducing functional coupling within the network, and the second where spontaneous activity of SST-INs is low and can be temporally synchronized to provide fast, feedback inhibition in the local network. Spontaneous activity of SST-INs thus reduces network activity in two separate ways: first, via direct and fast GABAaR-dependent inhibition of downstream targets and second, via slow and indirect suppression of local synaptic transmission.

The conditions that enable spontaneous activity of SST-INs, where they are a significant source of ambient GABA that silences not only local excitatory transmission between Pyr neurons^10^ but also excitatory synapses onto SST-INs, will be of great interest to identify. It remains unknown under what brain states presynaptic GABAbR are activated in SST-IN terminals in the neocortex; it is reasonable to hypothesize that this might happen during the states in which SST-IN activity is higher than during the quiet state.

Notably, GABAbR-mediated inhibition by SST-INs is not global, as synaptic efficacy between Pyr neurons and PV-INs was not changed with optogenetic suppression of SST-IN activity. The second important finding of our study shows that fast synaptic inhibition mediated by SST-INs on to local Pyr cells is not suppressed by the tonic activity of presynaptic GABAbRs. These data indicate that SST-INs might provide effective inhibition to the local network under high network activity conditions, in contrast to PV-INs which fast inhibition can be suppressed by tonic activity of GABAbRs. Future studies will reveal the specific cellular source of GABA responsible for activating GABAbR on SST- and PV-IN terminals.

Inhibitory interneurons play an essential role in controlling cortical activity at various temporal and spatial scales^42^. SST-INs preferentially inhibit distal parts of Pyr neurons whereas PV-INs synapse on the soma and proximal parts of the target neurons^3, 4, 43^, regulating inputs and outputs of excitatory neurons, respectively. Here, we provide a functional comparison for the sensitivity of two main neocortical interneurons in the activation of GABAbRs. Under conditions of high GABA release, the differential sensitivity of SST- and PV-INs to GABAbR modulation will favor fast (GABAaRs) inhibition mediated by SST- over PV-INs. Our data show that the weak excitatory synaptic input onto SST-INs from local Pyr cells can be further reduced by the spontaneous activity of SST-INs. However, SST-INs can still generate powerful GABAa-mediated inhibition thanks to their intrinsic spontaneous activity that is insensitive to GABAbRs.

Because Pyr neurons receive not only local inputs from neighboring Pyr neurons within L2/3 but also from other cortical layers^24, 25, 28, 44^ as well as other brain areas^45–47^, GABAbR-mediated silencing of local inputs might shift the balance of control of L2/3 Pyr neurons from local to long-range inputs. Consistent with this, prior studies have shown that thalamocortical inputs to L4 excitatory neurons are resistant to GABAbR modulation^48^ but see^39^. Thus, our data suggest that when SST-IN activity is high, local circuit amplification of incoming sensory information will be weak.

Pharmacological experiments for induction of synaptic plasticity in acute brain slices have yielded mixed results about the role of GABAbRs. For example, in the hippocampus, GABAbRs prevent the induction of long-term potentiation (LTP) in SST-INs^30^ but can facilitate the induction of LTP in CA1 Pyr cells^49, 50^, indicating that the effects of these receptors may be synapse-specific and also implicating these receptors in synaptic plasticity during learning and memory. In layer 4 (L4) of primary visual cortex of rats, the strengthening of synapses from FS cells (presumably PV-IN) to Pyr neurons requires the activity of postsynaptic GABAbRs, although LTP between L4 excitatory cells was not affected by GABAbR antagonists^51^. Critically, these pharmacological manipulations may not precisely recapitulate the tonic activation of GABAbRs under more naturalistic activity conditions. It remains unknown whether LTP at other excitatory synapses is sensitive to GABAbR activation, and which interneurons may regulate plasticity at input- and target-specific synapses. Interestingly, recent studies have reported that dually innervated dendritic spines on hippocampal pyramidal neurons are resistant to structural plasticity due to tonic inhibition of NMDA receptors through GABAbRs and SST-INs^52^.

Taken together, our data demonstrate that GABAbRs modulate neocortical networks in a neuron- and synapse-specific manner (Supplementary Fig. 2). The differential sensitivity of specific synapses and neurons to GABAbR-modulation may fine-tune the balance of excitation and inhibition in a compartment-specific manner, providing tight control of information flow to Pyr neurons in superficial layers.

## Materials and methods

### Ethical approval

All experimental procedures were conducted in accordance with the Act on the Protection of Animals Used for Scientific or Educational Purposes in Poland (Act of 15 January 2015, changed 17 November 2021; directive 2010/63/EU) and the National Institute of Health guidelines in USA. All experimental protocols were approved by Polish Ministry of Environment (Dec. No 47/2019) and the Institutional Animal Care and Use Committee at Carnegie Mellon University (Approval No PROTO201600045). The study was reported in accordance with ARRIVE guidelines.

### Animals

Mice were housed under controlled light cycles (12-h light–dark cycles) with ad libitum access to food and water.

The following strains of mice were used: (1) Sst-IRES-Cre mice on a C57Bl6 background (Jackson Labs stock # 013044); (2) Pvalb-T2A-Cre-D on a C57Bl6 background (Jackson Labs stock #012358); (3) PValb-tdTomato-miGAD67 (Jackson Labs stock #028594); (4) Ai14 mice on a mixed background, C57Bl6J and B6;129S6 (Jackson Labs stock #007908); (5) Ai35D on a C57Bl6 background (Jackson Labs stock # 012735). Experiments were performed in offspring of Sst-IRES-Cre mice crossed to either Ai14 (floxed-Tdt) or Ai35D (floxed-Arch-YFP) reporter mice, in offspring of Pvalb-T2A-Cre-D mice crossed to Ai14, in offspring of PValb-tdTomato-miGAD67 crossed to Sst-IRES-Cre. All transgenes were used as heterozygotes and both sexes were used.

### Virus injection

Virus injection procedures were performed at CMU and conducted with the NIH guidelines (USA) and approved by the Institutional Animal Care and Use at Carnegie Mellon University (Approval No PROTO201600045). New born (P0-1) double transgenic mice (Sst-IRES-cre:: PValb-tdTomato-miGAD67) were injected with a flex–GFP-ArchT virus to the somatosensory cortex to optogenetically silence SST-INs. Mouse newborns were anesthetized by incubation on ice prior to virus injection. The virus (rAAV1/flex-ArchT-GFP, UNC Vector Core) was delivered using a glass micropipette (tip diameter 10–20 μm) attached to a picospritzer microinjector (WPI). In vitro electrophysiology experiments were conducted 18–25 days after virus injection.

### Brain slice preparation

At the age of P18–P28, where P0 indicates the day of birth, mice were deeply anaesthetized with isoflurane and killed by decapitation using procedures in accordance with the Polish Animal Protection Act (Act of 15 January 2015, changed 17 November 2021; directive 2010/63/EU) and the NIH guidelines (USA).

Brain slices (350 μm thick) were prepared by an “across-row” protocol in which the anterior end of the brain was cut along a 45° plane toward the midline^53^. Slices were recovered and maintained at 24 °C in regular artificial cerebrospinal fluid (ACSF) composed of (in mM): 119 NaCl, 2.5 KCl, 2 MgSO_4_, 2 CaCl_2_, 1 NaH_2_PO_4_, 26.2 NaHCO_3_, 11 glucose equilibrated with 95/5% O_2_/CO_2_.

### Whole-cell recording

To enable spontaneous firing of SST-INs and tonic activation of GABAbRs, recordings were performed in modified ACSF (mACSF) solution composed of (in mM): 119 NaCl, 3.5 KCl, 0.5 MgSO_4_, 1 CaCl_2_, 1 NaH_2_PO_4_, 26.2 NaHCO_3_, 11 glucose equilibrated with 95/5% O_2_/CO_2_, as described before^10, 18^. To prevent the network from spontaneous firing, a part of experiments were performed in regular ACSF (rACSF) where the following ingredients were changed to 2.5 KCl, 1.3 MgSO_4_, 2.5 CaCl_2_.

Somata of L2/3 neurons in primary somatosensory cortex were targeted for whole-cell recording with borosilicate glass electrodes, resistance 4–8 MΩ. For current clamp mode, electrode internal solution was composed of (in mM): 125 potassium gluconate, 2 KCl, 10 HEPES, 0.5 EGTA, 4 Mg-ATP, and 0.3 Na-GTP, at pH 7.25–7.35, 290 mOsm and contained trace amounts of Alexa 488 to verify the location of the recorded cell. For voltage clamp mode, electrode internal solution was composed of (in mM): 130 cesium gluconate, 10 HEPES, 0.5 EGTA, 8 NaCl, 10 tetraethylammonium chloride, 5 QX-314, 4 Mg-ATP, and 0.3 Na-GTP, at pH 7.25–7.35, 290 mOsm and contained trace amounts of Alexa 488.

Because of the difficulty in identifying connected pairs, the majority of recordings were carried out at room temperature (24 °C) to enable longer recording periods, since prolonged incubation at warmer temperatures degrades cell health and diminished recording quality.

Electrophysiological data were acquired by Multiclamp 700A or Multiclamp 700B (Molecular Devices) and digitized with a National Instruments acquisition interface or Digidata 1550B (Molecular Devices). The data were filtered at 3 kHz, digitized at 10–20 kHz and collected by Igor Pro 6.0 (Wavemetrics) or pClamp (Molecular Devices). Series and input resistances were analyzed online, recordings were discarded when access or series resistances were unstable more than 30%.

### Neuron classification

Neurons were classified as Pyr neurons according to Pyr-like soma shape, the presence of an apical dendrite and spines visible after Alexa filling reconstruction as well as according to regular spiking in response to 500 ms suprathreshold intracellular current injection. SST-IN were identified using fluorescent reporter gene expression in Sst-Cre::Ai14, whereas PV-INs were visualized by the fluorescent marker genetically encoded in Pvalb-T2A-Cre-D::Ai14 or PValb-tdTomato-miGAD67 mice. Additionally, the firing responses to the somata current injection were analyzed. SST-INs responded to current steps with low threshold spiking (LTS) firing with a spike-rate adaptation, and the AHP after the first action potential was more negative than the last AHP during the current step^15^. PV-INs were identified by fast-spiking (FS) firing with a non-adapting firing pattern and AHP magnitudes that were uniform throughout a given current step.

### Connectivity analysis

Synaptically connected neurons were identified by paired whole-cell patch-clamp recordings. The distance between cells was  ≤ 300 μm. To evaluate Pyr to SST-IN connections (Pyr-SST) or Pyr to PV-IN (Pyr-PV), both cells were maintained in current clamp mode and EPSPs were recorded in SST- or PV-IN in response to the current injection to the presynaptic Pyr. Interneuron membrane potential was maintained at the hyperpolarized value about − 60 mV to block spontaneous spiking activity of the recorded cell and to visualize EPSPs. To evaluate inhibitory interneuron to Pyr connections, the membrane potential of an interneuron was maintained in current clamp, whereas the postsynaptic cell was kept in voltage clamp at the holding potential of 0 mV to record IPSCs in the response to the presynaptic spikes evoked by the current injection. Alternatively, IPSPs were recorded in current clamp mode at the depolarizing membrane potential of about − 55 mV. To assess connectivity, we analyzed responses from 20 trials delivered at 0.1 Hz of a 10-stimulus spike train (3–5 ms long, 1 nA at 20 Hz). The 10-spike train was critical for accurate assessment of synaptic connections, since weak and facilitating connections (such as at Pyr to SST-IN synapses) cannot be detected with single spikes^28^. Evoked postsynaptic responses were calculated using responses from 10 trials of the stimulus train. To precisely identify the onset of the synaptic response, analysis was focused on events that occurred during DOWN-states, since responses during UP-states were difficult to isolate from background activity in the sweep^54^. Because in vivo, L2/3 Pyr neurons rarely respond more than a single spike and almost never at frequencies exceeding 20 Hz^55^, we considered responses to the first presynaptic spike to be most representative of synaptic function as it might occur during normal sensory-evoked activity. Thus, amplitude and failure rate measurements are plotted for the EPSP (or IPSC/P) evoked by the first stimulus in the spike train.

Spontaneous excitatory postsynaptic currents (sEPSCs) were recorded in voltage clamp at − 70 mV. Spontaneous firing was recorded at the resting membrane potentials without any correction. Spontaneous activity was analyzed within at least 3 min of current-clamp recordings in control ACSF and ACSF with agents.

Intrinsic excitability was accessed using square pulses of 500 ms of increasing amplitude up to maximal firing frequency (steps of 10 pA and 20 pA, for SST- and PV-INs, respectively). To control for potential effects of GABAbR agents on Vrest, the membrane potential of interneurons was maintained at − 65 mV across different pharmacological conditions.

### Pharmacology

The GABAb receptor antagonist (CGP 55845, 1 μM) and agonist (baclofen, 10 μM), as well as the AMPAR antagonist (DNQX, 20 μM) and NMDAR antagonist (APV, 50 μM) were bath applied for at least 10 min before data acquisition to assess drug effect for 20 trails of the 10-pulse train. All the pharmacological agents were purchased from Tocris.

### Optical stimulation

For ArchT activation, photo stimulation was produced by a light-emitting diode (white LED with 590 nm filter set to maximum range, Prizmatix, Israel) and delivered through a 40 × water-immersion objective. After establishing a connection, SST-IN silencing was initiated 1 s or 1.5 s prior to the 10 pulse presynaptic train and maintained to the end of the spike train. Because the light hyperpolarized the membrane potential of SST-INs, the light ON trials were collected before light OFF trials (20 repetitions at 0.1 Hz for both periods). The light OFF trails were collected at the membrane potential of SST-INs adjusted by the constant somata current injection to the same value as it was during light ON period.

### Data analysis

Population data are presented as mean ± SD. 1–2 cells or 1 synaptically connected pair were analyzed in an individual mouse. Statistical significance was defined as *p* < 0.05 using a two-tailed paired t-test or Wilcoxon test depending on the normality distribution. The normality distribution was tested with the Shapiro–Wilk test and equal variance was analyzed with Brown-Forsythe test. For intrinsic excitability, a plot of the relation between the number of action potentials and the intensity of the injected current (I-F curve) was created for every neuron in control ACSF and after the drug application. The effect of a drug on excitability was analyzed as the difference in the rheobase, the maximal firing frequency and the difference of the spiking frequency at the same current steps in the comparison to control ACSF.

## Supplementary Information

Supplementary Figures.

## Data Availability

The data and material that support the findings of this study are available upon request to the corresponding author.

## References

[1] Rudy B, Fishell G, Lee S, Hjerling-Leffler J (2011). Three groups of interneurons account for nearly 100% of neocortical GABAergic neurons. Dev. Neurobiol..

[2] Wamsley B, Fishell G (2017). Genetic and activity-dependent mechanisms underlying interneuron diversity. Nat. Rev. Neurosci..

[3] Kawaguchi Y, Kubota Y (1997). GABAergic cell subtypes and their synaptic connections in rat frontal cortex. Cereb. Cortex.

[4] Wang Y (2004). Anatomical, physiological and molecular properties of Martinotti cells in the somatosensory cortex of the juvenile rat. J. Physiol..

[5] Kvitsiani D (2013). Distinct behavioural and network correlates of two interneuron types in prefrontal cortex. Nature.

[6] Pi H-J (2013). Cortical interneurons that specialize in disinhibitory control. Nature.

[7] Lovett-Barron M (2014). Dendritic inhibition in the hippocampus supports fear learning. Science.

[8] Gentet LJ (2012). Unique functional properties of somatostatin-expressing GABAergic neurons in mouse barrel cortex. Nat. Neurosci..

[9] Muñoz W, Tremblay R, Levenstein D, Rudy B (2017). Layer-specific modulation of neocortical dendritic inhibition during active wakefulness. Science.

[10] Urban-Ciecko J, Fanselow EE, Barth AL (2015). Neocortical somatostatin neurons reversibly silence excitatory transmission via GABAb receptors. Curr. Biol..

[11] Bowery NG (1981). Bicuculline-insensitive GABA receptors on peripheral autonomic nerve terminals. Eur. J. Pharmacol..

[12] Connors BW, Malenka RC, Silva LR (1988). Two inhibitory postsynaptic potentials, and GABAA and GABAB receptor-mediated responses in neocortex of rat and cat. J. Physiol..

[13] Deisz RA, Prince DA (1989). Frequency-dependent depression of inhibition in guinea-pig neocortex in vitro by GABAB receptor feed-back on GABA release. J. Physiol..

[14] Booker SA (2017). Differential surface density and modulatory effects of presynaptic GABA(B) receptors in hippocampal cholecystokinin and parvalbumin basket cells. Brain Struct. Funct..

[15] Fanselow EE, Richardson KA, Connors BW (2008). Selective, state-dependent activation of somatostatin-expressing inhibitory interneurons in mouse neocortex. J. Neurophysiol..

[16] Somjen GG (2004). Ions in the brain: Normal function, seizures, and stroke.

[17] Maffei A, Nelson SB, Turrigiano GG (2004). Selective reconfiguration of layer 4 visual cortical circuitry by visual deprivation. Nat. Neurosci..

[18] Urban-Ciecko J, Jouhanneau J-S, Myal SE, Poulet JFA, Barth AL (2018). Precisely timed nicotinic activation drives SST inhibition in neocortical circuits. Neuron.

[19] Sanchez-Vives MV, McCormick DA (2000). Cellular and network mechanisms of rhythmic recurrent activity in neocortex. Nat. Neurosci..

[20] Guy J, Möck M, Staiger JF (2023). Direction selectivity of inhibitory interneurons in mouse barrel cortex differs between interneuron subtypes. Cell Rep..

[21] Perez-Zabalza M (2020). Modulation of cortical slow oscillatory rhythm by GABA(B) receptors: An in vitro experimental and computational study. J. Physiol..

[22] Kaplanian A, Vinos M, Skaliora I (2022). GABA(B) - and GABA(A) -receptor-mediated regulation of Up and Down states across development. J. Physiol..

[23] Urban-Ciecko J, Barth AL (2016). Somatostatin-expressing neurons in cortical networks. Nat. Rev. Neurosci..

[24] Kapfer C, Glickfeld LL, Atallah BV, Scanziani M (2007). Supralinear increase of recurrent inhibition during sparse activity in the somatosensory cortex. Nat. Neurosci..

[25] Silberberg G, Markram H (2007). Disynaptic inhibition between neocortical pyramidal cells mediated by Martinotti cells. Neuron.

[26] Pala A, Petersen CCH (2015). In vivo measurement of cell-type-specific synaptic connectivity and synaptic transmission in layer 2/3 mouse barrel cortex. Neuron.

[27] Fino E, Yuste R (2011). Dense inhibitory connectivity in neocortex. Neuron.

[28] Jiang X (2015). Principles of connectivity among morphologically defined cell types in adult neocortex. Science.

[29] Booker SA (2020). Presynaptic GABA(B) receptors functionally uncouple somatostatin interneurons from the active hippocampal network. Elife.

[30] Booker SA (2018). Postsynaptic GABA(B)Rs inhibit L-type calcium channels and abolish long-term potentiation in hippocampal somatostatin interneurons. Cell Rep..

[31] Jouhanneau J-S, Kremkow J, Poulet JFA (2018). Single synaptic inputs drive high-precision action potentials in parvalbumin expressing GABA-ergic cortical neurons in vivo. Nat. Commun..

[32] Avermann M, Tomm C, Mateo C, Gerstner W, Petersen CCH (2012). Microcircuits of excitatory and inhibitory neurons in layer 2/3 of mouse barrel cortex. J. Neurophysiol..

[33] Lüscher C, Jan LY, Stoffel M, Malenka RC, Nicoll RA (1997). G protein-coupled inwardly rectifying K+ channels (GIRKs) mediate postsynaptic but not presynaptic transmitter actions in hippocampal neurons. Neuron.

[34] Kaupmann K (1998). GABA(B)-receptor subtypes assemble into functional heteromeric complexes. Nature.

[35] Degro CE, Kulik A, Booker SA, Vida I (2015). Compartmental distribution of GABAB receptor-mediated currents along the somatodendritic axis of hippocampal principal cells. Front. Synaptic. Neurosci..

[36] Sloviter RS, Ali-Akbarian L, Elliott RC, Bowery BJ, Bowery NG (1999). Localization of GABA(B) (R1) receptors in the rat hippocampus by immunocytochemistry and high resolution autoradiography, with specific reference to its localization in identified hippocampal interneuron subpopulations. Neuropharmacology.

[37] Liu Y, Yang XJ, Xia H, Tang C-M, Yang K (2019). GABA releases from parvalbumin-expressing and unspecific GABAergic neurons onto CA1 pyramidal cells are differentially modulated by presynaptic GABA(B) receptors in mouse hippocampus. Biochem. Biophys. Res. Commun..

[38] Shao C (2022). Presynaptic GABA(B) receptors differentially modulate GABA release from cholecystokinin and parvalbumin interneurons onto CA1 pyramidal neurons: A cell type-specific labeling and activating study. Neurosci. Lett..

[39] Kruglikov I, Rudy B (2008). Perisomatic GABA release and thalamocortical integration onto neocortical excitatory cells are regulated by neuromodulators. Neuron.

[40] Takesian AE, Kotak VC, Sharma N, Sanes DH (2013). Hearing loss differentially affects thalamic drive to two cortical interneuron subtypes. J. Neurophysiol..

[41] Liguz-Lecznar M, Urban-Ciecko J, Kossut M (2016). Somatostatin and somatostatin-containing neurons in shaping neuronal activity and plasticity. Front. Neural Circuits.

[42] Isaacson JS, Scanziani M (2011). How inhibition shapes cortical activity. Neuron.

[43] Kuljis DA (2019). Fluorescence-based quantitative synapse analysis for cell type-specific connectomics. eNeuro.

[44] Lefort S, Petersen CCH (2017). Layer-dependent short-term synaptic plasticity between excitatory neurons in the C2 barrel column of mouse primary somatosensory cortex. Cereb. Cortex.

[45] Kawaguchi Y, Shindou T (1998). Noradrenergic excitation and inhibition of GABAergic cell types in rat frontal cortex. J. Neurosci..

[46] Kinnischtzke AK, Simons DJ, Fanselow EE (2014). Motor cortex broadly engages excitatory and inhibitory neurons in somatosensory barrel cortex. Cereb. Cortex.

[47] Chen N, Sugihara H, Sur M (2015). An acetylcholine-activated microcircuit drives temporal dynamics of cortical activity. Nat. Neurosci..

[48] Gil Z, Connors BW, Amitai Y (1997). Differential regulation of neocortical synapses by neuromodulators and activity. Neuron.

[49] Davies CH, Starkey SJ, Pozza MF, Collingridge GL (1991). GABA autoreceptors regulate the induction of LTP. Nature.

[50] Mott DD, Lewis DV (1991). Facilitation of the induction of long-term potentiation by GABAB receptors. Science.

[51] Wang L, Maffei A (2014). Inhibitory plasticity dictates the sign of plasticity at excitatory synapses. J. Neurosci..

[52] Kleinjan MS (2022). Dually innervated dendritic spines develop in the absence of excitatory activity and resist plasticity through tonic inhibitory crosstalk. Neuron.

[53] Finnerty GT, Roberts LS, Connors BW (1999). Sensory experience modifies the short-term dynamics of neocortical synapses. Nature.

[54] Steriade M, Timofeev I, Grenier F (2001). Natural waking and sleep states: A view from inside neocortical neurons. J. Neurophysiol..

[55] Barth AL, Poulet JFA (2012). Experimental evidence for sparse firing in the neocortex. Trends Neurosci..

