# Supplementary material for: Somatostatin-expressing interneurons modulate neocortical network through GABAb receptors in a synapse-specific manner
Source: Sci Rep. 2023 May 31;13:8780. doi: 10.1038/s41598-023-35890-2 (PMC10232538; doi:10.1038/s41598-023-35890-2)
Supplement: Supplementary file 1 — Supplementary Figures. [file 41598_2023_35890_MOESM1_ESM.pdf]

Supplementary Figure 1

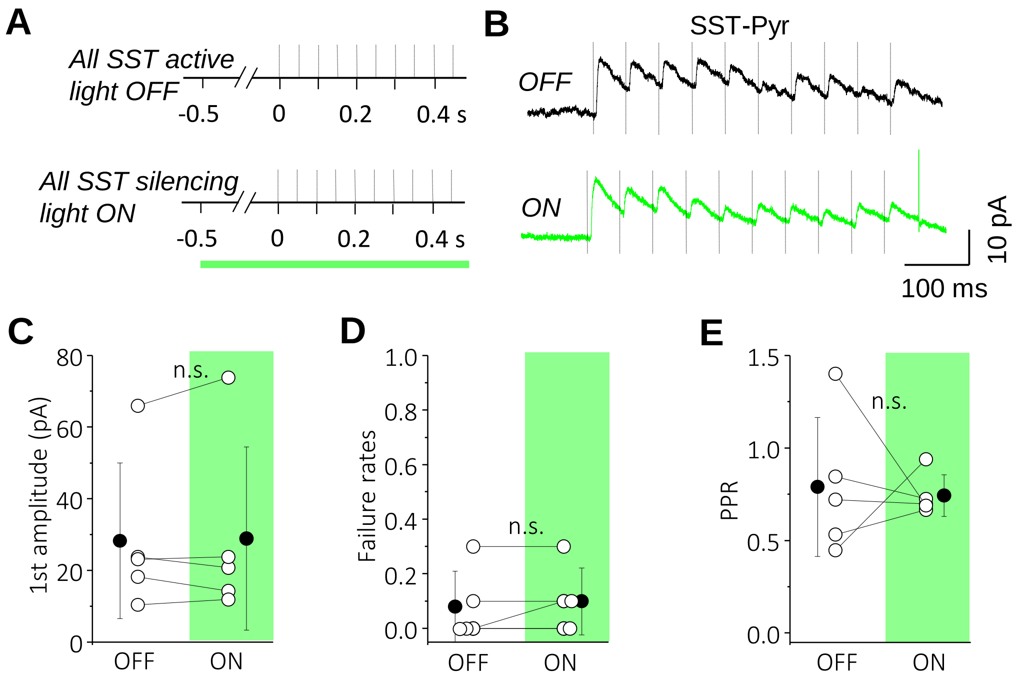

Supplementary Figure 1. SST-IN spontaneous firing does not activate GABAbRs at SST-Pyr synapses. (A) Schematic of the stimulation protocol. 1-single green light (1s) was started 0.5 s prior to the presynaptic spike train. **(B)** The averaged trace of IPSC under baseline/light OFF and light ON conditions. **(C)** With-in cell comparison and mean (±SD) IPSC amplitude in response to the first spike in the train, for both conditions (paired t-test, n.s. p=0.787, n=5 cells). **(D)** The same as (C) but for failure rates after the first spike, for both conditions (paired t-test, n.s. p=0.374, n=5 cells). **(E)** The same as (C) but for PPR, for both conditions (paired t-test, n.s. p=0.827, n=5 cells).

Supplementary Figure 2

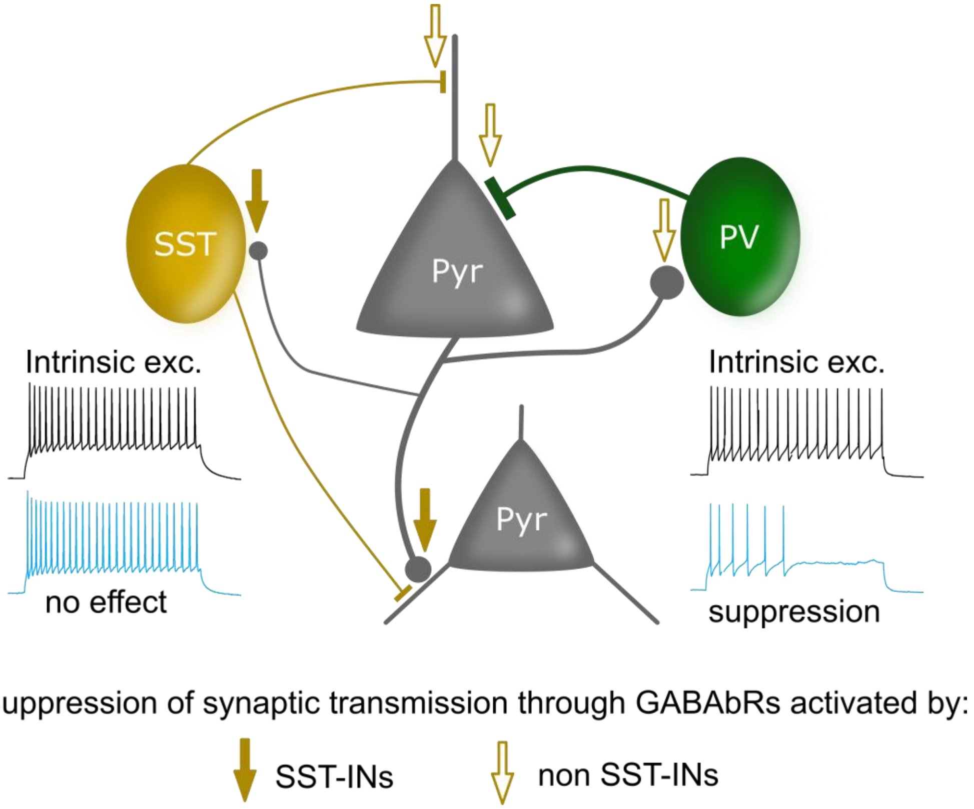

Supplementary Figure 2. GABAbRs modulate the L2/3 microcircuit in a synapse- and cell-specific manner. SST-INs activate GABAbRs onto synapses between pyramidal neurons and pyramidal cells to SST-INs (solid arrows). SST-INs do not activate GABAbRs onto SST- and PV-IN outputs and PV-IN excitatory inputs (open arrows). PV- but not SST-IN intrinsic excitability is controlled by GABAbRs.
